# Arachidonic acid suppresses hepatic cell growth through ROS‐mediated activation of transglutaminase

**DOI:** 10.1002/2211-5463.12511

**Published:** 2018-09-11

**Authors:** Xian‐Yang Qin, Jun Lu, Muyi Cai, Soichi Kojima

**Affiliations:** ^1^ Liver Cancer Prevention Research Unit RIKEN Center for Integrative Medical Sciences Wako Japan; ^2^ Beijing Engineering Research Center of Protein & Functional Peptides China National Research Institute of Food and Fermentation Industries Beijing China

**Keywords:** arachidonic acid, corn peptides, hepatic cell injury, nuclear transglutaminase 2, reactive oxygen species

## Abstract

We previously reported a profound augmentation in the hepatic levels of a pro‐inflammatory precursor, arachidonic acid (AA), during liver tumorigenesis. Here, we report a critical role of the induced reactive oxygen species (ROS)‐mediated cellular activation of a protein cross‐linking enzyme, transglutaminase 2 (TG2), in liver injury by AA. In cultures of hepatic cells, AA dose‐dependently suppressed cell growth, which accompanied the induced nuclear accumulation of TG2, as demonstrated in EGFP‐tagged, TG2‐overexpressing hepatic cells. A chemical inhibitor/shRNA that acts against TG2 prevented AA‐mediated cell growth suppression. In addition, AA provoked significant production of ROS, and antioxidants blocked AA‐induced activation of nuclear TG2 and hepatic cell growth suppression. We propose that AA‐mediated oxidative stress and TG2 transamidase activity might contribute to chronic liver injury and inflammation and thereby serve as potential therapeutic targets for the chemoprevention of hepatocellular carcinoma.

**Abbreviations**
5‐BAPA: 5‐biotinamidopentylamine
AA: arachidonic acid
CMH2DCFDA: chloromethyl derivative of 2′,7′‐dichlorodihydrofluorescein diacetate
CP: corn oligopeptide
DEN: diethylnitrosamine
EGFR: epidermal growth factor receptor
FAs: fatty acids
GAPDH: glyceraldehyde 3‐phosphate dehydrogenase
HCC: hepatocellular carcinoma
HO‐1: heme oxygenase 1
NAC: N‐acetyl‐L‐cysteine
NASH: nonalcoholic steatohepatitis
NF‐κB: nuclear factor‐kappa B
Nrf2: nuclear factor erythroid‐2‐related factor 2
ROS: reactive oxygen species
shRNA: short hairpin RNA
TG2: transglutaminase 2
ZDON: Z‐DON‐Val‐Pro‐Leu‐OMe

Lipogenesis in the liver is induced abundantly in individuals with nonalcoholic steatohepatitis (NASH) and chronic hepatitis virus infection and is associated with a high risk and poor prognosis of NASH‐related hepatocellular carcinoma (HCC) 1. For example, it is reported that fatty acids (FAs) released from fat‐accumulated hepatocytes induce an increase in the production of mitochondrial‐derived reactive oxygen species (ROS) and cause cell death in intrahepatic CD4^+^ T lymphocytes, which leads to the loss of immune surveillance and accelerated NASH‐driven hepatic tumorigenesis 2. Another important cause of lipogenesis in the liver, which contributes to the emergence of HCC, is repeated liver injury and compensatory proliferation due to inflammation 3. Hence, blocking lipogenesis using the chemical inhibitor metformin has been reported as an effective therapeutic strategy for preventing liver tumorigenesis 4.

One of the well‐documented events that is induced under altered lipogenesis in the liver is the production of arachidonic acid (AA); this compound is the main pathogen‐like unsaturated FA that mediates inflammation following tumor development 5. We previously found that hepatic AA contents were increased during tumorigenesis in a hepatocellular carcinogen [diethylnitrosamine (DEN)]‐induced hepatic tumorigenesis model and that acyclic retinoid, a chemopreventive agent against HCC 6, 7, prevented this augmentation of AA in obese mice 8. Consistent with our results, elevated levels of serum AA have been observed in HCC patients 9. AA is recognized as the main factor mediating repeated liver injury and compensatory proliferation. However, the mechanism underlying the induction of liver injury by AA has not yet been fully elucidated.

Transglutaminase 2 (TG2) is the most ubiquitously expressed member of the TG family; members of this family cross‐link substrate proteins 10. We previously reported that the nuclear accumulation of TG2 in hepatic cells 11 resulted in the cross‐linking and inactivation of Sp1 transcription factor, thereby reducing the expression of Sp1‐responsive genes such as *c‐Met* and epidermal growth factor receptor (EGFR); these genes are essential for survival of cells, and the reduction in their expression leads to cellular apoptosis 12. Suppression of TG2 activity partially prevented cell death 13. The enhanced expression of both nuclear TG2 and cross‐linked Sp1 was also evident in the livers of the patients with alcoholic steatohepatitis (ASH) 14 and in those with NASH 14. However, the mechanism underlying the activation of nuclear TG2 in the liver of ASH/NASH patients remains unclear. Very recently, in a coculture system of pathogenic fungi and hepatic cells, we found that fungi‐derived ROS, such as hydroxyl radicals, play a critical role in nuclear TG2‐dependent liver injuries 15.

Given that mitochondrial ROS play an important role in AA toxicity 16, we hypothesized that the induction of AA in ROS might also mediate hepatic cell death through the induction of nuclear TG2. In this study, we explored this hypothesis and obtained *in vitro* evidence that the suppression of hepatic cell growth by AA accompanies ROS production and the activation of nuclear TG2. A chemical inhibitor/shRNA that acts against TG2 attenuated the suppressed hepatic cell growth by AA, and importantly, the blockade of ROS production prevented the AA‐induced nuclear TG2 activation and growth suppression in the hepatic cells.

## Materials and methods

### Chemicals

AA (A9673), the ROS inhibitor *N*‐acetyl‐l‐cysteine (NAC; A7250), and a nuclear TG2 inhibitor, phenosafranin (199648), were obtained from Sigma‐Aldrich (St. Louis, MO, USA). An irreversible TG inhibitor, Z‐DON‐Val‐Pro‐Leu‐OMe (ZDON; Z006), was purchased from Zedira (Darmstadt, Germany). The oligopeptide fraction was prepared from spray‐dried corn protein powder (Zhucheng Xingmao Corn Developing Co., Ltd., Zhucheng, China) as previously described 15.

### Cell culture

JHH7 cells, a HCC cell line, were kindly supplied by Matsuura of the Jikei University School of Medicine, Tokyo, Japan 17. The cells were maintained in Dulbecco's modified Eagle's medium (Wako Industries, Osaka, Japan) containing 10% fetal bovine serum (Mediatech, Herndon, VA, USA) containing 100 U·mL^−1^ penicillin/streptomycin and 2 mmol·L^−1^ l‐glutamine (Mediatech) and were grown at 37 °C in a humidified incubator under 5% CO_2_.

### Cell viability assay

Cell viability was measured using a Cell Counting Kit‐8 (Dojindo Molecular Technologies, Tokyo, Japan); the absorbance was measured using a plate reader (ARVO MX; Perkin Elmer Inc., Waltham, MA, USA) at 450 nm 8.

### Immunofluorescence analysis

Cells were seeded in Greiner 96‐well microtiter plates (Greiner Bio‐One), fixed in 4% paraformaldehyde for 10 min, and then incubated in 0.5% Triton X‐100 in PBS for 10 min at room temperature as previously reported 7. After the cells were blocked in 5% fetal bovine serum/TPBS for 1 h at room temperature, they were incubated overnight at 4 °C with primary antibodies, including mouse anti‐TG2 (1 : 200, ab2386; Abcam, Cambridge, MA, USA), rabbit anti‐nuclear factor erythroid‐2‐related factor 2 (Nrf2) (1 : 400, ab62352; Abcam), mouse anti‐Ki67 (1 : 200, 350502; BioLegend, San Diego, CA, USA), or control rabbit/mouse IgG. The cells were then washed and stained with fluorochrome (FITC/TRITC)‐conjugated secondary antibodies (1 : 500; Invitrogen, Eugene, OR, USA) for 20 min at room temperature. Cell nuclei were visualized using DAPI (1 : 2000; Wako Industries). Cellular fluorescence signals were detected using an ImageXpressMICRO High‐Content Screening System (Molecular Devices, Sunnyvale, CA, USA). Morphological analysis was performed using the ‘Multi‐Wavelength Cell Scoring Application Module’ in MetaXpress Image Analysis software (Molecular Devices).

### Determination of *in vitro* ROS production

ROS production was analyzed based on the incorporation of the chloromethyl derivative of 2′,7′‐dichlorodihydrofluorescein diacetate (CMH2DCFDA; Life Technologies) (2.5 μm) for 30 min at 37 °C. After chemical treatment for the indicated time, the cells were monitored for their FITC fluorescence signals using a plate reader (ARVO MX; Perkin Elmer Inc.) or an ImageXpressMICRO High‐Content Screening System (Molecular Devices).

### RNA isolation and real‐time RT‐PCR

Total RNA was isolated using an RNeasy Kit (Qiagen, Valencia, CA, USA) and quantified using a NanoDrop spectrophotometer (NanoDrop products). cDNA was synthesized using a PrimeScript RT Master Mix Kit (TaKaRa Bio, Otsu, Japan). The sequences of the primers used were as follows (5′ to 3′): glyceraldehyde 3‐phosphate dehydrogenase (GAPDH) forward (CAATGACCCCTTCATTGACC) and reverse (GACAAGCTTCCCGTTCTCAG); TG2 forward (CCTTA CGGAGTCCAACCTCA) and reverse (CCGTCTTCTGCT CCTCAGTC); and heme oxygenase 1 (HO‐1) forward (AACTTTCAGAAGGGCCAGGT) and reverse (CTGGG CTCTCCTTGTTGC). PCRs were performed using a Roche LightCycler 96 Real‐Time PCR System (Roche Diagnostic Co., Ltd.) and the SYBR Premix ExTaq II (TaKaRa Bio).

### Determination of *in vitro* TG activity

Cells were seeded in a 96‐well plate, and the cellular activity of TG was measured based on the incorporation of 0.2 mm 5‐biotinamidopentylamine (5‐BAPA, 21345; Thermo Fisher Scientific, Rockford, IL, USA) into the cells, which were incubated in the presence of 0.1 mm aminoguanidine for chemical treatment as described elsewhere 15. The cells were then fixed with 4% paraformaldehyde, blocked, and immunostained with streptavidin/TRITC (1 : 500, 016‐020‐084; Jackson ImmunoResearch Laboratories, West Grove, PA, USA). The TG activity was then detected as TRITC fluorescence, which was analyzed using an ImageXpressMICRO High‐Content Screening System (Molecular Devices).

### Transduction of shRNA lentiviral particles

TG2 (sc‐37514‐v) and control (sc‐108080) short hairpin RNA (shRNA) lentiviral particles were obtained from Santa Cruz Biotechnology (Santa Cruz, CA, USA). Cells were seeded in a 12‐well plate and cultured until they reached approximately 50% confluence. The cells were then transduced with lentiviral vectors expressing shRNA at approximately 1 multiplicity of infection (MOI) using 5 μg·mL^−1^ Polybrene (sc‐134220; Santa Cruz Biotechnology) for 24 h. Clones stably expressing the shRNA were selected using 2 μg·mL^−1^ puromycin‐containing culture medium.

### Plasmid and transfection

The expression vector for the enhanced GFP (EGFP)‐tagged full‐length TG2 (GFP‐TG2) was amplified from TG2‐pSG5 vector and ligated into the pEGFP‐C1 vector (Clontech Laboratories, Inc., Palo Alto, CA, USA) as reported elsewhere 11. The cells were transfected with mock or GFP‐TG2 plasmids using Lipofectamine 2000 (0.1 μg of DNA/0.5 μL of Lipofectamine 2000; Invitrogen). GFP fluorescence was detected and analyzed using an ImageXpressMICRO High‐Content Screening System (Molecular Devices).

### Statistical analysis

Quantitative data are expressed as the mean ± SD of at least three replicates. The significance of differences between values was assessed using Student's *t*‐test. Values of *P* < 0.05 were considered to indicate significance.

## Results and Discussion

### AA suppressed cell growth in the hepatic cell cultures

**Figure 1:**
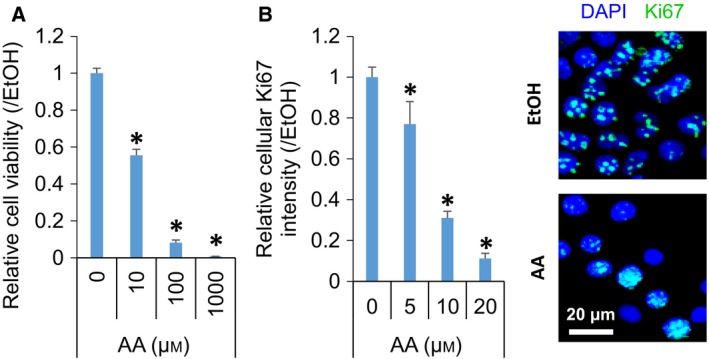
AA suppressed cell growth in hepatic cells. Dose‐dependent decrease in relative cell viability (A) and the ratio (left panel) and representative images (right panel) of Ki67‐positive proliferating cells in JHH7 cells treated with AA for 24 h (B). Scar bar: 20 μm. The data are presented as the mean (*n* = 3–4 replicates) ± SD; *P < 0.05, Student's *t*‐test.

The cytotoxic effect of AA was observed in the studied hepatic cells. AA dose‐dependently decreased both cell viability (Fig. 1A) and the expression of a cellular proliferation marker, Ki67 (Fig. 1B).

### AA induced the nuclear location of TG2, and the suppression of TG2 activity prevented the AA‐suppressed cell growth in the hepatic cells

**Figure 2:**
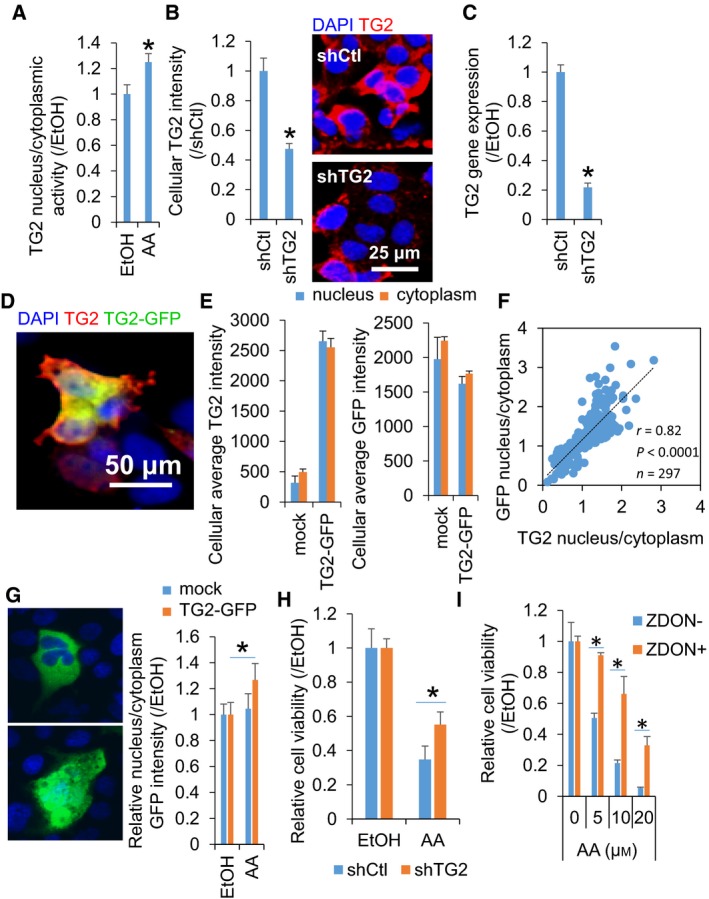
The suppression of TG2 activity prevented AA‐suppressed growth in hepatic cells. (A) Quantitative data regarding the nucleus/cytoplasm activity of TG2 in JHH7 cells treated with 20 μm AA for 24 h. The cellular activity of TG2 was measured as fluorescence intensity resulting from the TRITC‐based incorporation of 5‐BAPA. (B) Quantitative data (left panel) and representative images (right panel) of immunofluorescence staining and (C) gene expression for TG2 in JHH7 cells transduced with control (shCtl) or TG2 (shTG2) shRNA lentiviral particles. Scar bar: 25 μm. (D ~ H) JHH7 cells whose endogenous TG2 was knocked down with shTG2 and in which GFP‐TG2 was overexpressed (GFP‐TG2‐transfected shTG2 JHH7 cells). (D) A representative image of immunofluorescence staining for TG2. Scar bar: 50 μm. (E) Changes in cellular TG2 levels in the cells after treatment with 5 μm AA for 16 h. Cellular TG2 and GFP intensities in mock‐ or GFP‐TG2‐transfected shTG2 JHH7 cells. Data are presented as fluorescent intensities in the nucleus and cytoplasm, separately. (F) Correlation between the nucleus/cytoplasm intensity ratios of TG2 and GFP in the GFP‐TG2‐transfected shTG2 JHH7 cells. (G) The enhanced nuclear translocation of TG2 in the GFP‐TG2‐transfected shTG2 JHH7 cells upon treatment with 5 μm AA for 16 h. Scar bar: 10 μm. (H) The cell viability of shCtl and shTG2 JHH7 cells upon treatment with 10 μm AA for 24 h. (I) The cell viability of JHH7 cells upon treatment with increasing concentrations of AA (as indicated) in the absence or presence of 50 μm ZDON for 24 h. The data are presented as means (*n* = 3–4 replicates) ± SD; *P < 0.05, Student's *t*‐test.

Next, we examined whether the growth suppression by AA in the hepatic cell cultures might be associated with the TG2‐dependent pathway. Treatment of JHH7 cells with AA enhanced the activity of nuclear TG2, as measured by the incorporation of a TG substrate, 5‐BAPA (Fig. 2A). This phenomenon can be explained in two ways: AA increased the nuclear translocation of TG2 and/or induced the transamidase activity of TG2 in the nucleus. Since the nuclear accumulation of TG2 has been shown to be a critical step in the TG2‐mediated apoptosis of hepatic cells 12, we investigated whether AA might affect the subcellular location of TG2 in the hepatic cells. To avoid the effect of endogenous TG2, the cells were transduced with TG2 shRNA lentiviral particles in which endogenous TG2 was stably knocked down by 80% (Fig. 2B,C) and in which GFP‐TG2 was overexpressed in its place (Fig. 2D) 11. Immunofluorescence analysis using a monoclonal anti‐TG2 antibody showed a dramatic increase in intracellular TG2 intensity in the GFP‐TG2‐overexpressing cells compared to mock‐transfected cells, while no marked difference was observed in GFP intensity (Fig. 2E). The cellular localization of TG2 appeared to be heterogeneous within the cell populations in a dish, probably due to differences in their cell cycle phase, and a strong correlation between the nucleus/cytoplasm intensity ratios of TG2 and GFP was observed in the GFP‐TG2‐overexpressing cells (Fig. 2F). Together, these data showed that our methodology was appropriate to evaluate the subcellular location of TG2. The results showed that AA (5 μm) enhanced the nuclear accumulation of TG2 by 27% in the GFP‐TG2‐overexpressing hepatic cells (Fig. 2G). Nuclear TG2 serves as a cross‐linking enzyme and promotes apoptosis 12. Therefore, we examined whether the inhibition of TG2 activation might prevent the growth‐suppressing effect of AA in the studied hepatic cells. The results showed that AA‐mediated cell growth suppression was partly prevented by the knockdown of TG2 using its shRNA (Fig. 2H) and by the suppression of TG2 activity using the irreversible TG2 inhibitor, ZDON 18 (Fig. 2I). shTG2 and ZDON prevented 31% and 57%, respectively, of the growth suppression resulting from the treatment with 10 μm AA for 24 h. In addition, phenosafranin, which inhibits the nuclear localization of TG2 without affecting its transamidase activity 19, also significantly inhibited the AA‐induced cell growth suppression in JHH7 cells (Fig. S1).

### Blockade of ROS production prevented AA‐induced nuclear TG2 activation and growth suppression in hepatic cells

**Figure 3:**
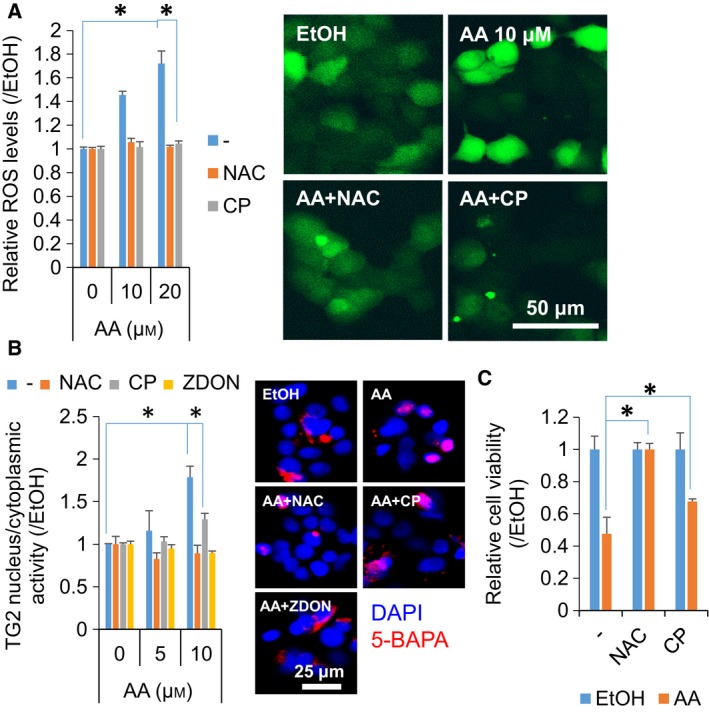
Blockade of ROS production prevented the AA‐induced activation of nuclear TG2 and cell growth suppression. (A) Quantitative data (left panel) and representative images (right panel) of the CMH2DCFDA staining of JHH7 cells upon treatment with increasing concentrations of AA (as indicated) in the absence or presence of 5 mm NAC or 0.5 mg·mL^−1^ CP for 24 h. Scar bar: 50 μm. (B) Quantitative data (left panel) and representative images (right panel) of the nucleus/cytoplasm activity of TG2 in JHH7 cells upon treatment with increasing concentrations of AA (as indicated) in the absence or presence of 5 mm NAC, 0.5 mg·mL^−1^ CP, or 50 μm ZDON for 24 h. Scar bar: 25 μm. The cellular activity of TG2 was measured as fluorescence intensity resulting from the TRITC‐based incorporation of 5‐BAPA. (C) The cell viability of JHH7 cells treated with 5 μm AA in the absence or presence of 10 mm NAC or 0.5 mg·mL^−1^ CP for 24 h. The data are presented as means (*n* = 3 replicates) ± SD; *P < 0.05, Student's *t*‐test.

Finally, we examined whether the growth‐suppressing effect of AA on the hepatic cells might be associated with the production of ROS. AA dose‐dependently induced ROS production, as detected using CMH2DCFDA, in the hepatic cells, and this was significantly blocked by an ROS scavenger, NAC, and a food‐derived natural antioxidant, corn oligopeptide (CP), to basal levels (Fig. 3A). Next, we examined the effect of AA on the activation of Nrf2, a master transcription factor that is activated in response to ROS; Nrf2 becomes stable in the cytoplasm and then translocates into the nucleus in response to ROS to regulate the expression of antioxidant genes 20. Immunofluorescence analysis showed that AA significantly induced the nuclear translocation of Nrf2 in the studied hepatic cells (Fig. S2A). Consistently, the gene expression of HO‐1, a downstream target of Nrf2, was also markedly induced by AA (Fig. S2B). We previously reported that *Candida albicans* killed hepatic cells through the ROS‐mediated activation of nuclear TG2 15, a finding that prompted us to examine whether the ROS produced by AA treatment might also induce the activation of nuclear TG2 and consequent cell growth suppression. The results showed that the AA‐induced activation of nuclear TG2 was significantly prevented by the antioxidants NAC and CP; this prevention occurred at similar levels to those obtained using the irreversible inhibitor of TG2, ZDON, suggesting that AA induced the nuclear TG2 activity through the production of ROS (Fig. 3A,B). Finally, we found that the blockade of ROS production by NAC and CP significantly prevented the growth‐suppressing activity of AA in the hepatic cells (by 100 and 38%, respectively, against 10 μm AA) (Fig. 3C). We propose that AA‐mediated oxidative stress and TG2 transamidase activity might contribute to chronic liver injury and inflammation and thereby serve as potential therapeutic targets for the chemoprevention of HCC.

## Conclusion

**Figure 4:**
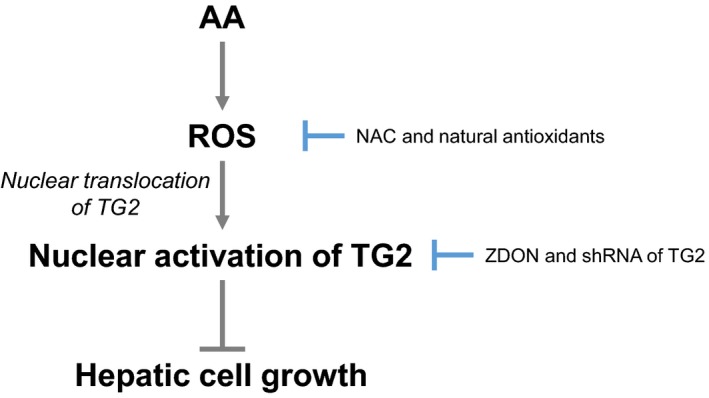
A schematic model of a ROS‐mediated TG2‐dependent signaling pathway underlying the AA‐induced growth suppression of hepatic cells.

We demonstrate here that AA suppressed the cell growth of hepatic cells by inducing the production of ROS and its mediated cellular TG2 activation (Fig. 4). AA stimulated ROS production and subsequently increased cellular (mostly nuclear) TG2 transamidase activity, partly by promoting the nuclear translocation of TG2. Blocking AA‐induced ROS production and TG2 activation using chemical agents or natural nutrients efficiently prevented AA‐suppressing cell growth.

## Author contributions

XYQ and SK conceived and designed the experiments and wrote the manuscript. XYQ performed the experiments and analyzed the data. JL and MC contributed new reagents/analytic tools. MC and SK supervised the research. All authors were involved in the discussion and editing of the final version of the manuscript.

## Supporting information


**Fig. S1.** A TG2 inhibitor repressed the AA‐induced growth suppression of hepatic cells. The cell viability of JHH7 cells treated with increasing concentrations of AA (as indicated) in the absence or presence of 30 μm phenosafranin (an inhibitor that blocks the nuclear localization of TG2) for 24 h. The data are presented as means (*n* = 4 replicates) ± SD; *P < 0.05, Student's *t* test.
**Fig. S2.** AA activated the Nrf2 pathway in hepatic cells. (A) Representative images (left panel) and quantitative data (right panel) regarding the nuclear translocation of Nrf2 and (B) HO‐1 gene expression in JHH7 cells upon treatment with 5 μm AA for 24 h. Scar bar: 25 μm. The data are presented as means (*n* = 3 replicates) ± SD; *P < 0.05, Student's *t* test.
