# Supplementary material for: Arachidonic acid suppresses hepatic cell growth through ROS‐mediated activation of transglutaminase
Source: FEBS Open Bio. 2018 Sep 11;8(10):1703–10. doi: 10.1002/2211-5463.12511 (PMC6168684; doi:10.1002/2211-5463.12511)
Supplement: Supplementary file 2 [file FEB4-8-1703-s002.docx]

**Supplemental Figure Legends**

**Fig. S1. A TG2 inhibitor repressed the AA-induced growth suppression of hepatic cells.** The cell viability of JHH7 cells treated with increasing concentrations of AA (as indicated) in the absence or presence of 30 μM phenosafranin (an inhibitor that blocks the nuclear localization of TG2) for 24 h. The data are presented as means (n = 4 replicates) ± SD; **P* < 0.05, Student’s *t* test.

**Fig. S2. AA activated the Nrf2 pathway in hepatic cells.** (A) Representative images (left panel) and quantitative data (right panel) regarding the nuclear translocation of Nrf2 and (B) HO-1 gene expression in JHH7 cells upon treatment with 5 μM AA for 24 h. Scar bar: 25 μm. The data are presented as means (n = 3 replicates) ± SD; **P* < 0.05, Student’s *t* test.
